# An outbreak of norovirus-associated acute gastroenteritis associated with contaminated barrelled water in many schools in Zhejiang, China

**DOI:** 10.1371/journal.pone.0171307

**Published:** 2017-02-07

**Authors:** Xiaopeng Shang, Xiaofei Fu, Peng Zhang, Minyang Sheng, Jianqiang Song, Fan He, Yinwei Qiu, Haocheng Wu, Qinbao Lu, Yan Feng, Junfen Lin, Enfu Chen, Chengliang Chai

**Affiliations:** 1 Department of Public Health Surveillance and Advisory, Zhejiang Provincial Center for Disease Control and Prevention, Hangzhou, Zhejiang, China; 2 Department of Communicable Disease Control and Prevention, The Center for Disease of Jiaxing City, Jiaxing, Zhejiang,China; 3 Department of Center Office, The Center for Disease Control and Prevention of Huzhou City, Huzhou, Zhejiang, China; 4 Department of Center Office, The Center for Disease Control and Prevention of Haining County, Jiaxing, Zhejiang, China; 5 Department of Center Office, The Center for Disease Control and Prevention of Haiyan County, Jiaxing, Zhejiang, China; Institut Pasteur of Shanghai Chinese Academy of Sciences, CHINA

## Abstract

**Objectives:**

More than 900 students and teachers at many schools in Jiaxing city developed acute gastroenteritis in February 2014. An immediate epidemiological investigation was conducted to identify the pathogen, infection sources and route of transmission.

**Methods:**

The probable cases and confirmed cases were defined as students or teachers with diarrhoea or vomiting present since the term began in February 2014. An active search was conducted for undiagnosed cases among students and teachers. Details such as demographic characteristics, gastrointestinal symptoms, and drinking water preference and frequency were collected via a uniform epidemiological questionnaire. A case-control study was implemented, and odds ratios (ORs) and 95% confidence intervals were calculated. Rectal swabs from several patients, food handlers and barrelled water factory workers, as well as water and food samples, were collected to test for potential bacteria and viruses.

**Results:**

A total of 924 cases fit the definition of the probable case, including 8 cases of laboratory-confirmed norovirus infection at 13 schools in Jiaxing city between February 12 and February 21, 2014. The case-control study demonstrated that barrelled water was a risk factor (OR: 20.15, 95% CI: 2.59–156.76) and that bottled water and boiled barrelled water were protective factors (OR: 0.31, 95% CI: 0.13–0.70, and OR: 0.36, 95% CI: 0.16–0.77). A total of 11 rectal samples and 8 barrelled water samples were detected as norovirus-positive, and the genotypes of viral strains were the same (GII). The norovirus that contaminated the barrelled water largely came from the asymptomatic workers.

**Conclusions:**

This acute gastroenteritis outbreak was caused by barrelled water contaminated by norovirus. The outbreak was controlled after stopping the supply of barrelled water. The barrelled water supply in China represents a potential source of acute gastroenteritis outbreaks due to the lack of surveillance and supervision. Therefore, more attention should be paid to this area.

## Introduction

Norovirus (NoV) is known as a major cause of acute nonbacterial gastroenteritis among adults and causes several acute gastroenteritis (AGE) outbreaks every year. It can be classified into six genogroups (GI-GVI) according to the characteristics of the genome. GI and GII are responsible for diseases in humans; moreover, GIV can also infect humans but is rarely checked out investigated”[1–4]. A systematic review found that the median incubation periods of the GI and GII gene groups were 1.1 days (95% CI: 1.1–1.2) and 1.2 days (95% CI: 1.1–1.2 days), respectively, with no significant differences [5]. Most cases are mild, and the symptoms are characterized by diarrhoea and vomiting, followed by nausea, abdominal cramps, headache, fever, chills, and muscle aches, all of which might typically disappear after 2–3 days without treatment. Some elderly and young children may develop intense symptoms, resulting even in death. A few cases may also be asymptomatic infections, and the proportion of asymptomatic norovirus infection during epidemic season is higher than in the popular season [6]. Norovirus infection has obvious wintertime seasonality. NoV infection is often associated with food contaminated by microorganisms, and according to relevant literature reports, more than 50% of food-borne disease outbreaks are caused by food contaminated with the NoV [7].

China currently lacks a surveillance system regarding NoV gastroenteritis; however, many outbreaks of NoV gastroenteritis in schools have been reported in recent years [8–10]. Unlike the outbreaks of NoV gastroenteritis associated with person-to-person transmission (62%) and contaminated food (14%) reported in the foreign literature [11], numerous outbreaks of NoV gastroenteritis in China have occurred in collective units such as schools and kindergartens and were mainly attributed to contaminated water. Sixteen NoV AGE outbreaks were reported from 2003 to 2013 through the Emergent Public Health Event Information Management System (EPHEIM) in Zhejiang province; most outbreaks (14/16) were caused by contaminated barrelled water.

**Fig 1:**
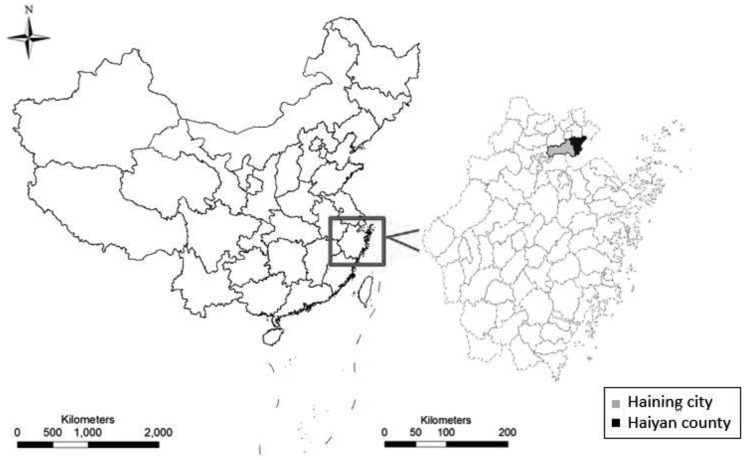
The geographic distribution of NoV AGE that occurred in Jiaxing, China, February 12–21, 2014.

On February 17, 2014, The Center for Disease Control and Prevention of Jiaxing City (JXCDC) was notified that hundreds of students at many schools located in Haining city and Haiyan county (Fig 1) had developed symptoms of diarrhoea and vomiting of unknown cause; the event attracted media attention. We immediately formed a team to conduct an epidemiological investigation to determine the cause of the pathogen, infection sources, route of transmission and risk factors.

## Materials and methods

### Epidemiological investigation

In this outbreak, the study population consisted of probable cases and confirmed cases. Probable cases were defined as teachers and students from all types of schools in the Jiaxing region with the symptoms of diarrhoea (≥three times/day accompanied by variation in stool properties) or vomiting after the beginning of the term (February 2014). Confirmed cases were those probable cases that tested positive for norovirus via reverse transcription polymerase chain reaction (RT-PCR). An active search was conducted for cases among all students and teachers in all types of schools and kindergartens in this region; the cases were searched based on a uniform epidemiological questionnaire. Data were obtained either face-to-face or by calling the patients’ parents regarding demographic characteristics (gender, age, school, grade, class), gastrointestinal symptoms (nausea, vomiting, diarrhoea, abdominal pain, fever), duration of illness and hospitalization.

To determine the potential transmission mode as soon as possible, we designed a case-control study to analyse the possible risk factors by using 69 cases and 70 controls, which were selected randomly from the same school for February 18. A questionnaire was designed to collect more information regarding drinking water preference and frequency, eating in the school cafeteria or not, personal hygiene habits, eating habits, and history of contact with a person with diarrhoea and/or vomiting. Drinking water preference included barrelled water (boiled or not), commercial bottled water or municipal tap water. The study members were interviewed face-to-face directly by the CDC investigators. Written informed consent was obtained from all participants, and the program was approved by the Ethics Committee of Zhejiang Provincial Centre for Disease Control and Prevention.

### Environmental hygiene investigation

We investigated the cafeteria’s hygiene conditions, health certificates of food handlers, drinking water situation and environmental hygiene conditions around those schools that presented cases. A further investigation of the environmental, production flow and disinfection conditions of the involved barrelled water factory was done, in which we found the suspected source of infection through the present case-control study.

### Specimen collection and laboratory tests

Rectal swabs, stool or vomitus specimens of the cases; rectal swabs of school cafeteria food handlers and workers at the barrelled water factory; food scraps from the school cafeteria; and drinking-water samples, including barrelled water, other drinking–water produced by the same factory and the raw water, were collected to detect intestinal viruses, including *Norovirus*, *Adenovirus* and *Rotavirus*, using RT-PCR, and intestinal bacteria, including *Salmonella*, *Shigella*, *enterohaemorrhagic Escherichia coli*, *Campylobacter*, *Staphylococcus aureus*, *Bacillus cercus*, and *Vibrio parahaemolyticus*, by culture. Drinking water samples were collected to detect *total bacterial count*, *total coliforms* and *thermotolerant coliforms*.

### Statistical analysis

The distributions of the major symptoms in the outbreaks are summarized based on frequencies and per cents. Chi-square tests of the attack rates in different groups were performed using SPSS software version 19.0, and the risk of this outbreak, odd ratios (ORs), and 95% confidence intervals were calculated. All statistical tests were 2-sided and *p* values <0.05 were considered to be statistically significant.

## Results

**Fig 2:**
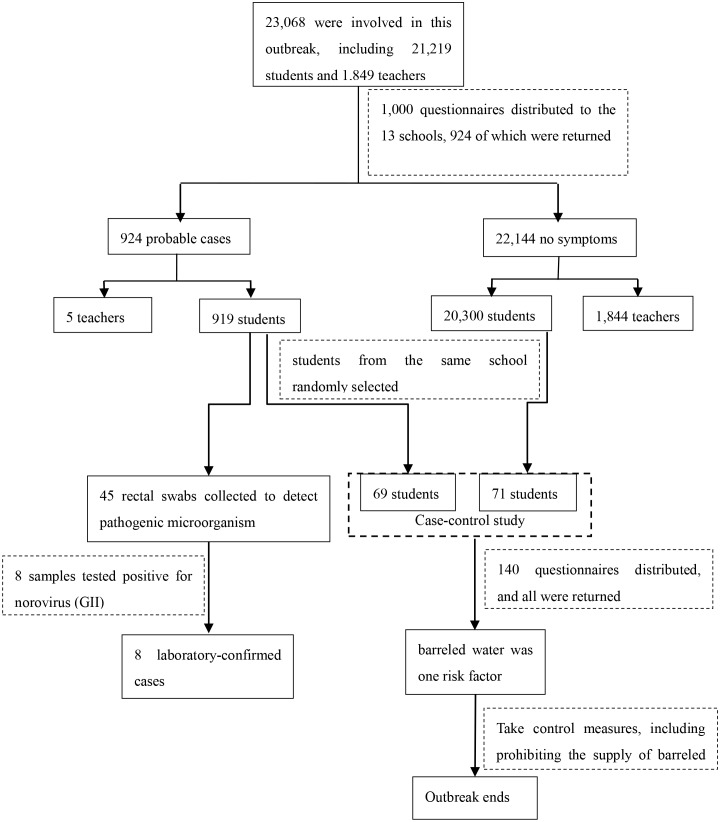
A brief flow-diagram of the NoV AGE that occurred in Jiaxing, China, February 12–21, 2014.

A total of 924 cases fit the definition of the probable case, including 8 cases of laboratory-confirmed norovirus infection between February 12 and February 21, 2014; the attack rate was 4.01%. Six middle schools in Haining city and 4 middle schools and 3 kindergartens in Haiyan county were involved. The attack rate for students was much higher than that for teachers. The highest and lowest attack rates for students were 10.47% and 1.22%, respectively. The grade and class distributions of the student cases were dispersed; in the schools, almost every grade had cases, with most of those grades having no more than 10 cases. A total of 11 rectal samples and 8 barrelled water samples were detected as norovirus-positive. The genotypes of the viral strains were the same (GII) in the patients and in the barrelled water. The JXCDC used immediate control measures, including surveillance, disinfection and prohibition of the barrelled water supply, to stop this outbreak (Fig 2).

### Descriptive epidemiology

The 924 cases included 919 students and 5 teachers (Table 1). The 919 student cases included 420 boys and 499 girls (sex ratio was 1:1.19), 564 boarding students and 355 non-boarding students; and the median age was 17 years old (range was 4–21 years old). The attack rates for middle school students, kindergarten students and teachers were significantly different (p<0.001). Among the 924 cases, 826 (89.39%) suffered from vomiting, 524 (56.71%) from nausea, 298 (32.25%) from abdominal pain, 206 (22.29%) from diarrhoea, 122 (13.20%) from fever and 129 (13.96%) from both vomiting and diarrhoea. No patients were hospitalized.

**Table 1:** Attack rates of the outbreak across different demographics.

characteristics	No. of cases	Total	Attack rate (%)	Chi-square	*p*-value
**Overall**	924	23068	4.01		
**Sex**					
Male	420	9910	4.24	0.387	0.534
Female	499	11309	4.41		
**Occupation**					
Middle school students	786	19934	3.94	210.817	0.000
Kindergarten students	133	1285	10.35		
Teachers	5	1849	0.27		
**Regional distribution**					
Haining	451	12126	3.72	5.448	0.020
Haiyan	473	10942	4.32		
**Boarding status (only students)**					
Boarding	564	12669	4.45	1.107	0.293
Not boarding	355	8550	4.15		

**Fig 3:**
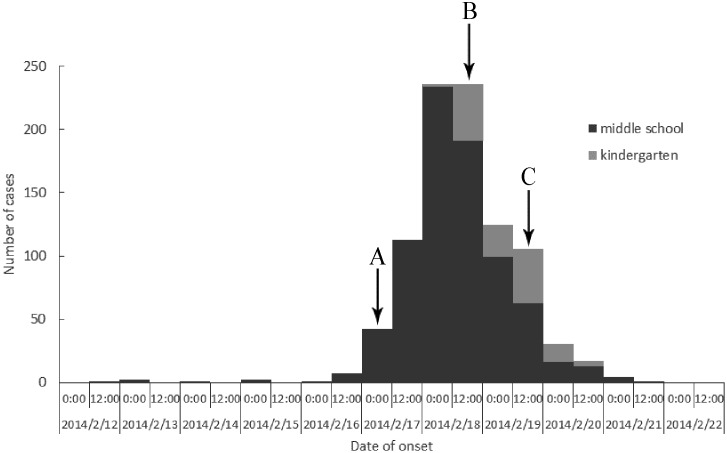
Time distribution of the onset of probable outbreak cases in 13 schools in Jiaxing, China, February 12–21, 2014: epidemic curve with 12 h intervals. A: Field epidemiological investigation B: Implementation of the case-control study C: Supply of barrelled water stopped.

The first case of gastroenteritis occurred on February 12 at 20:00. With the new semester starting on February 16, a rapid increase in the number of cases occurred over the following 3 days. The time distribution showed only one peak incidence on February 18 (Fig 3); no further cases were reported after February 21, 15:00.

### Case-control study

There were 69 probable cases and 70 control subjects recruited from different classes of the same middle school on February 18; they all replied to the questionnaires (response rate 100%). Univariate analysis results are summarized in Table 2. Compared with control subjects, barrelled water supplied by schools was one risk factor, while bottled water and boiled barrelled water were protective factors.

**Table 2:** Risk factors of the norovirus acute gastroenteritis outbreak.

	Cases(N = 69)		Controls(N = 70)		Odds ratio(95% CI)
	Yes N(%)	No N(%)	Yes N(%)	No N(%)	
Barrelled water	69(100.00)	0(0)	54(77.14)	16(22.86)	20.15(2.59–156.76)
Bottled water	59(85.51)	10(14.49)	25(35.71)	45(64.29)	0.31(0.13–0.70)
Boiled water from home	2(2.90)	67(97.10)	4(5.71)	66(94.29)	0.49(0.09–2.78)
Boiled barrelled water	16(23.19)	53(76.81)	25(35.71)	45(64.29)	0.36(0.16–0.77)
Share cups	14(20.29)	55(79.71)	11(15.71)	59(84.29)	0.46(0.30–1.72)
Closest classmate got diseased	52(75.36)	17(24.64)	63(90.00)	7(10.00)	3.66(0.96–6.11)
Saw vomiting	21(30.44)	48(69.56)	16(22.86)	54(77.14)	1.12(0.31–1.41)
Touched vomit	7(10.14)	62(89.86)	4(5.71)	66(94.29)	0.98(0.15–1.89)

### Laboratory tests

A total of 19 samples tested positive for norovirus (GII) based on RT-PCR, including 11 rectal swabs and 8 barrelled water samples. Of the 11 rectal swabs, 8 were from student cases and 3 were from the staff of the barrelled water factory. Total coliforms were observed in 56 water samples; 14 samples exceeded 100 colony-forming units/ml, which is the upper limit allowed by the Standard of Water Quality of China. All food samples were found to be negative for *Salmonella*, *Shigella*, *enterohaemorrhagic Escherichia coli*, *Campylobacter*, *Staphylococcus aureus*, *Bacillus cercus*, *Vibrio parahaemolyticus*, *Adenovirus* and *Rotavirus* (Table 3).

**Table 3:** Testing results of the samples taken from cases, staff, barrelled water and food during the norovirus acute gastroenteritis outbreak.

Sample	No.	Testing item	Result
Rectal swabs of the cases	45	NoV*	8 positive
Vomitus of the cases	1	NoV	Negative
Rectal swabs of cafeteria staff	40	NoV	All negative
Rectal swabs of barrelled water factory staff	19	NoV	3 positive
Unopened barrelled water remaining at school	12	NoV	1 positive
Remaining unopened barrelled water in schools and at supplier	18	Total coliforms	7 disqualified
Unopened barrelled water stored in barrelled water factory	4	NoV	1 positive
Unopened barrelled water selling on market	7	NoV/Total coliforms	1 positive/7 unqualified
Used barrelled water in schools	11	NoV	5 positive
Tap water	1	Total coliforms	Qualified
Raw barrelled water	3	NoV	All negative
Food samples from the school cafeteria	33	NoV/Adenovirus/Rotavirus/Other intestinal bacteria	All negative

*: NoV = Norovirus.

### Environmental hygiene investigation

#### Food survey

All 13 schools that had acute gastroenteritis patients had cafeterias, among which the middle school cafeterias mainly provided lunch and dinner every day, while the kindergarten cafeterias only supplied lunch every day. The hygiene condition of all the cafeterias was good and conformed to the management standard. Every cafeteria operated independently and no procurement, processing or selling of the same food together occurred. The types of food sold by those cafeterias were disparate, and salad and cold food were not found. No staff from the cafeterias developed gastroenteritis.

#### Drinking water survey

Through further investigation, we found that all 13 schools used barrelled water supplied by the same supplier, which was one of the specific pure water suppliers authorized by local education authorities. The barrelled water was produced by factories A and B. The average class uses approximately 2–3 barrels of barrelled water per day in every school, and students mostly drank water that was not boiled.

In factory A, the source of barrelled water was one large natural reservoir located in an ecological reserve. Before the production of drinking water, the water was drained into an artificial reservoir, which is located at the south-west corner of the factory. There was a large amount of garbage in the ladders that led to the artificial reservoir, with wood shavings and other debris floating on the water. The drinking water production process was as follows: raw water was stored in a storage barrel, filter processed in an airtight device, and sent to the filling zone. The plastic buckets, after washing, scrubbing in an automatic cleaning machine, steady-state chlorine dioxide (concentration 2.0–2.5%) disinfection, and rinsing using treated water, were sent to the filling area, followed by filling, adding a cap and sealing. Regarding factory B, an approximately 4 km straight line distance away from factory A, the source water was another natural reservoir. We could not determine its production process because it was shut down when we investigated.

A total of 4 water samples from unopened barrelled water stored in the factory were collected for detection; 1 sample produced by factory A tested positive for norovirus. Seven barrelled water samples from unopened barrelled water selling on the market were collected; 1 sample produced by factory B tested positive for norovirus. Nineteen rectal swabs from 19 workers of these two barrelled water factories were collected; 3 samples, including 1 from A and 2 from B, were positive for norovirus. The number of diarrhoea patients did not increase abnormally among the community residents during the outbreak in the schools and barrelled water factories, based on our further investigation of medical institutions located in Haining and Haiyan.

This indicates that barrelled water was likely the culprit of this outbreak, with water samples from the two factories testing positive for norovirus. In our survey, we obtained information showing that the barrelled water workers usually drank boiled water, and few directly drank barrelled water. Combined with the above survey, we can infer that there are two possible reasons for the barrelled water to be contaminated: one possibility is that the source water was not healthy and that the water processing method was simple and crude; the other possibility is that the staff directly washed their hands in the buckets, which probably left some pathogenic bacteria in the buckets that then contaminated the barrelled water.

### Control measures

We took the following preventative and control measures to stop the outbreak as soon as possible. First, morning check and illness-induced absence were implemented, which were enhanced by epidemic surveillance and reports. Second, the food, drinking water and environmental health at all schools were strictly ensured, along with thorough disinfection of a school when cases appeared. Third, all schools suspended the supply of barrelled water immediately and provided sufficient drinking water. Fourth, all patients were mandatorily taken home for treatment and returned to school 3 days after recovery, and the entire school was closed for at least 3 days if more than half of the students in one class were ill. Fifth, the students were educated regarding individual sanitation, and self-protection consciousness was improved. After a week of effort, this outbreak ended, with no new cases occurring after February 21st.

## Discussion

The results of this epidemiologic investigation indicated that the outbreak of acute gastroenteritis in many schools in 2 counties (cities) of Jiaxing city, Zhejiang province, China, was caused by the contamination of barrelled water by norovirus. (1) The epidemic curve showed one peak, which fits the characteristics of point source exposure. (2) All 13 schools purchased barrelled water for students’ daily drinking. The barrelled water was all supplied by the same supplier and produced by the same factories. The brand and batch of the water was the same or similar. Moreover, we detected norovirus in both opened and unopened barrelled water samples, as well as in rectal swabs from the barrelled water factory workers. (3) The rectal swab samples from the student patients and the barrelled water workers were investigated, and barrelled water samples were analysed, testing positive for NoV GII based on RT-PCR. (4) Although all subjects involved in the outbreak were housed in the schools and barrelled water factories, no risk factor was identified other than the contact with the barrelled water. In particular, no correlation was found with the consumption of any food. No cases of acute gastroenteritis were reported among the population of the nearby village during the same period. (5) The outbreak was quickly quelled after the cessation of the barrelled water supply and the implementation of thorough disinfection, which corroborated the epidemiological data and suggested that NoV-contaminated barrelled water was the source of the outbreak.

During this outbreak, the positive test outcome of so many barrelled water samples was the first to have been described in China, although several waterborne outbreaks have been described and associated with norovirus contamination in the drinking water in institutional settings [9,12]. Before extracting the viral RNA, all water samples were concentrated via membrane adsorption-elution in combination with the PEG precipitation method [13]. It was unfortunate that viral RNA from the NoV-positive barrelled water samples was not available for genomic sequence testing. We could not determine whether sequence homology of the NoV was identifiable in patients and barrelled water, although they were all classified as GII viruses.

Norovirus is increasingly recognized as an important pathogen of acute gastroenteritis. All age groups are vulnerable to this virus, but the majority of morbidity and mortality occurs at the extremes of age [14]. During this outbreak, the morbidity of kindergarteners was higher than in middle school students and teachers, which can likely be attributed to the higher chances of contact with each other for kindergarteners than for other people. Thus, there may be other routes of transmission in addition to drinking barrelled water for kindergarteners, e.g., person to person via the faecal-oral route, via aerosolized vomit, or from the contaminated environment via physical contact or aerosols [15]. Because the kindergartener cases all came from Haiyan county, the morbidity of Haiyan was higher than in Haining. Based on the shape of the epidemiological curve and laboratory testing outcomes, we identified that the contaminants largely came from the asymptomatic workers, regarding the samples positive for GII norovirus and that GI norovirus genotypes exhibit a higher resistance and longer persistence in the environment than GII [16].

Along with rapid economic development and deteriorating environmental condition in China, the supply of barrelled water is increasing, especially in schools. The risk of gastroenteritis outbreaks still exists because the quality of the commercially available barrelled water is generally poor [17,18]. The barrelled water supply in China represents a potential source of acute gastroenteritis outbreaks due to a lack of surveillance and supervision. The safety and quality of drinking water have become growing concerns among the general public, but this has not been well addressed. Thus, control measures should be strengthened to stop outbreaks such as this. In the Standard of Water Quality of China (GB5749-2006), there are no regulations on virus level. Revision and improvement of the standard is necessary. Moreover, more rapid, effective, and sensitive detection methods for various pathogens in drinking water should be further studied. Moreover, enhancement of the surveillance of infectious disease symptoms and timely discovery and control of disease outbreaks are critical measures to reduce the impact of such events.

Some limitations of this study should be noted. First, there may be many cases we did not identify because this outbreak was large-scale and the number of investigators was limited, which can lead to the underestimation of attack rate [19–22]. Second, to stop the outbreak as soon as possible, we implemented a case-control study with a smaller population that was selected randomly. We were therefore unable to fully investigate the transmission mode of this outbreak except for barrelled water. Third, we also could not draw conclusions based on the RT-PCR outcome because further genomic sequences testing was missing.

## Supporting information

S1 Questionnaire Cases questionnaire of the outbreak of norovirus infection. (PDF)
