# Supplementary material for: An outbreak of norovirus-associated acute gastroenteritis associated with contaminated barrelled water in many schools in Zhejiang, China
Source: PLoS One. 2017 Feb 7;12(2):e0171307. doi: 10.1371/journal.pone.0171307 (PMC5295720; doi:10.1371/journal.pone.0171307)
Supplement: S1 Questionnaire — (PDF) [file pone.0171307.s001.pdf]

知情同意书

您好，按照《中华人民共和国传染病防治法》要求，当地疾病预防控制中心对感染性腹泻病例需进行流行病学个案调查，并采集血液、肛拭子等相关标本开展病原学检测，这对于疫情的早期诊断以及暴发疫情的及时控制具有重要意义。

这是一份知情同意书，用来征求您的同意，本次流行病学调查将同时利用您的粪便标本，需要采集您的肛拭子开展病原学检测。在您决定是否同意前，我将向您介绍本次调查的有关情况。

调查目的：浙江省疾病预防控制中心和嘉兴市疾病预防控制中心将负责本次调查，该项调查的目的主要是为了了解此次感染病腹泻暴发原因，及时查找病原菌，为当地防治策略的制定提供科学依据。

要求：我们需要采集您的肛拭子标本开展病原学检测，希望得到您的同意和配合。如果您同意我们将通过调查表询问一些与该项调查有关的问题。

利益：此项调查近期可能不会使您受益。但您和别人将来会从调查结果中受益。

保密：您的个人记录和粪便样本将以一个编号来识别，我们对您的调查记录进行保密。您不会在任何有关该项调查的出版物中被暴露身份。

拒绝参加或退出调查的权利：参加此项调查完全出于自愿，您可以选择不参加，无论您的决定是什么，对您的利益都没有影响。

花费：本次调查无需您支付任何费用。

您的粪便病原学检测若成阳性，我们将通知您。

您对调查有问题，请与浙嘉兴市疾病预防控制中心 富小飞医生联系，电话：0573-83685307

签名：如果您对调查有问题，或者请他人阅读并解释给您听，而且您理解了上述信息，并自愿参加，请在下面签名或按手印。

参加者姓名：________________　　证人（监护人）姓名：________________

时间：_____年_____月_____日

诺如病毒感染聚集性和暴发疫情个案调查表

编号 □□□□□

一、基本情况

1、被调查对象类别：（1）疑似病例 （2）临床诊断病例 （3）实验室诊断病例

2、患者姓名：___________ 被访家长/家属姓名：_____________

3、性别： （1）男 （2）女

4、出生日期： ______年______月（年龄：_____周岁）

5、工作单位/学校：________________

6、工作部门/班级/班组：_____________ 入住宿舍则宿舍房间号：________

7、职业： （1）学生 （2）教师 （3）厨工，岗位______ （4）医护人员

（5）工人 （6）农民 （7）散居儿童 （8）幼托儿童 （9）其他

8、文化程度：（1）学龄前儿童 （2）文盲 （3）小学 （4）初中

（5）高中或中专 （6）大专及以上 （7）不详

9、现住址：_____________________

10、联系电话：_______________________

二、发病及诊疗经过

1、发病时间：____月_____日______时（上午/下午）

2、是否就诊： ①是 ②否 ③不清楚

首次就诊时间：____月_____日______时（上午/下午）

3、是否住院： ①是 ②否 ③不清楚

入院时间：____月_____日______时（上午/下午）

出院时间：____月_____日______时（上午/下午）

4、是否补液治疗：（1）口服补液治疗 （2）静脉补液治疗 （3）无

5、是否已经痊愈：①是 ②否 ③不清楚

痊愈时间：____月_____日______时（上午/下午）

三、临床表现

1、首发症状：

（1）腹泻　①是，_____次　②否　③不清楚

（2）腹痛　①是　②否　③不清楚

（3）恶心　①是　②否　③不清楚

（4）呕吐　①是，_____次　②否　③不清楚

（5）发热　①是，_____℃　②否　③不清楚

（6）其他____________________________

2、整个病程的临床表现：

症状体征	是否出现	开始出现时间	消失时间
腹泻	①是（最多___次/天）②否 ③不清楚	___月___日___时	___月___日___时
腹痛	①是　②否 ③不清楚	___月___日___时	___月___日___时
恶心	①是　②否 ③不清楚	___月___日___时	___月___日___时
呕吐	①是（最多___次/天）②否 ③不清楚	___月___日___时	___月___日___时
发热	①是（最高___℃）②否 ③不清楚	___月___日___时	___月___日___时
头痛	①是　②否 ③不清楚	___月___日___时	___月___日___时
肌肉酸痛	①是　②否 ③不清楚	___月___日___时	___月___日___时
其他		___月___日___时	___月___日___时
其他		___月___日___时	___月___日___时

3、临床检验结果

（1）首次血常规：采血时间：___月___日，结果：WBC_____（10^9/L）

血小板_____（10^9/L）

（2）第二次血常规：采血时间：___月___日，结果：WBC_____（10^9/L）

血小板_____（10^9/L）

（3）首次便常规：采样时间：___月___日，结果：WBC 计数_____

RBC 计数_____　病毒检测结果___________

（4）第二次便常规：采样时间：___月___日，结果：WBC 计数_____

RBC 计数_____　病毒检测结果___________

四、流行病学：

1、宿舍/家庭同住______人，发病______人（不含患者本人）

同班或同部门______人，发病______人（不含患者本人）

填写其他人员发病情况（根据实际情况定是否需要填下表）：

姓名	性别	年龄	发病时间（具体到小时）	接触方式	与患者关系	联系方式

注：性别：（1）男　（2）女；

接触方式：（1）同吃　（2）同住　（3）一起上学　（4）一起工作

（5）同活动　（6）其他：__________

2、发病前 72 小时内同类病人暴露情况

2.1 是否接触同类病人：

接触方式：（1）同吃　（2）同住　（3）一起上学　（4）一起工作

（5）同活动　（6）其他__________

首次接触同类病人时间：___月___日___时（指病人发病后的首次暴露）

最后接触同类病人时间：___月___日___时

接触时是否采取防护：（1）是，__________　（2）否

2.2 是否直接接触过患者呕吐物或粪便：

（1）是　　（2）否，跳至 2.3　（3）不清楚，跳至 2.3

首次接触时间：___月___日___时

最后接触时间：___月___日___时

接触时是否采取防护：（1）是，__________　　（2）否

2.3 是否短距离暴露过患者呕吐物或粪便（一米内）：（1）是　　（2）否

首次接触时间：___月___日___时

最后接触时间：___月___日___时

接触时是否采取防护：（1）是，__________　　（2）否

3、发病前 72 小时内摄入的食物（包括食品、饮料、酒和水果等）

略

4、发病前 72 小时内饮水史

4.1 是否喝生水：（1）是　　（2）否

4.2 生活用水来源：（1）自来水　（2）井水　（3）河水　（4）泉水　（5）开水

（6）桶装水，品牌__________　（7）瓶装水，品牌__________　（8）其它

4.3 饮水来源：（1）自来水　（2）井水　（3）河水　（4）泉水　（5）开水

（7）桶装水，品牌__________　（7）瓶装水，品牌__________　（8）其它

5、个人卫生

5.1 饭前便后洗手：（1）每次都洗　（2）有时洗手（3）偶尔洗手　（4）从不洗手

5.2 是否用洗手液或肥皂：（1）是　（2）否

5.3 是否喜爱吃生冷食：　（1）是　（2）否

6、其他情况：____

____

____

调查员：__________　　调查时间：______年______月______日
